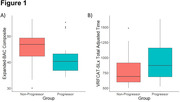# Utility of two computerized assessments for predicting clinical progression in individuals with early Alzheimer’s Disease

**DOI:** 10.1002/alz.089032

**Published:** 2025-01-03

**Authors:** Dorothee Schoemaker, Alexandra Atkins, Chelsea Abraham, Matthew Welch, Brenda L Plassman, Nancy Sickel, Corrine Madsen, Hans Klein, Danielle T DiGregorio, Kathleen A. Welsh‐Bohmer, Richard SE Keefe

**Affiliations:** ^1^ WCG, Princeton, NJ USA; ^2^ Duke University Medical Center, Durham, NC USA

## Abstract

**Background:**

Interventional trials in Alzheimer’s Disease (AD) are increasingly targeting early disease stages. To optimize the likelihood of successful outcomes for these trials, there is an important need for tools capable of identifying individuals prone to exhibit clinical progression throughout the course of the trial. The Expanded Brief Assessment of Cognition (Expanded‐BAC) and the Virtual Reality Functional Capacity Assessment Tool – Short List (VRFCAT‐SLx) are two computerized tests assessing cognitive and functional abilities, respectively. They offer automated instructions and scoring, which significantly improves standardization and reduces administration errors. Importantly, they minimize reliance on caregiver availability, which can be a significant barrier to trial participation. The goal of this project was to evaluate the value of the Expanded‐BAC and VRFCAT‐SLx in predicting clinical progression in individuals with early AD.

**Method:**

A total of 51 participants with clinical diagnoses of MCI or early AD (NIA‐AA clinical criteria) completed both a baseline and follow‐up visit including the CDR, Expanded‐BAC, and VRFCAT‐SLx. The mean follow‐up interval was of 9.5 months (SD = 4.7 months). Clinical progression was operationalized as a change of 0.5 or more on the CDR Sum of Boxes score (CDR SoB) over the follow‐up interval. Baseline performance on the Expanded‐BAC and the VRFCAT‐SLx was contrasted between clinical progressors and non‐progressors using ANCOVAs adjusting for follow‐up duration. Linear regression models were computed to assess the predictive value of baseline performance on the Expanded‐BAC and the VRFCAT‐SLx on CDR SoB changes over time, also adjusting for follow‐up duration.

**Result:**

At follow‐up, there were 18 clinical progressors and 33 non‐progressors. The Expanded‐BAC composite score and the VRFCAT‐SLx total adjusted time at baseline significantly differed between progressors and non‐progressors, with progressors showing poorer performance on both measures (Figure 1). In linear regression models, performance at baseline on the Expanded‐BAC (r^2^ = 0.24, p<0.01) and the VRFCAT‐SLx (r^2^ = 0.16, p<0.05) significantly predicted changes in CDR SoB over the follow‐up interval.

**Conclusion:**

These preliminary results suggest that performance on the Expanded‐BAC and the VRFCAT‐SLx can predict clinical progression in individuals with early AD. These two computerized tests might thus be useful tools to identify individuals likely to exhibit clinical progression in the course of a trial.